# Estimating submarine groundwater discharge in Jeju volcanic island (Korea) during a typhoon (Kong-rey) using humic-fluorescent dissolved organic matter-Si mass balance

**DOI:** 10.1038/s41598-020-79381-0

**Published:** 2021-01-13

**Authors:** Hyung-Mi Cho, Tae-Hoon Kim, Jae-Hong Moon, Byung-Chan Song, Dong-Woon Hwang, Taejin Kim, Dong-Hoon Im

**Affiliations:** 1 Department of Ocean Sciences, Inha University, 100 Inha-ro, Incheon, 22212 Republic of Korea; 2 Department of Oceanography, Faculty of Earth Systems and Environmental Sciences, Chonnam National University, Gwangju, 61186 Republic of Korea; 3 Department of Earth and Marine Sciences, Jeju National University, Jeju, 63243 Republic of Korea; 4 Marine Environment Research Division, National Institute of Fisheries Science, Busan, 46083 Republic of Korea; 5 Department of Oceanography, Pukyong National University, 45 Yongso-ro, Nam-gu, Busan, 48513 Republic of Korea

**Keywords:** Biogeochemistry, Ocean sciences, Marine chemistry

## Abstract

We examined the residence time, seepage rate, and submarine groundwater discharge (SGD)-driven dissolved nutrients and organic matter in Hwasun Bay, Jeju Island, Korea during the occurrence of a typhoon, Kong-rey, using a humic fluorescent dissolved organic matter (FDOM_H_)-Si mass balance model. The study period spanned October 4–10, 2018. One day after the typhoon, the residence time and seepage rate were calculated to be 1 day and 0.51 m day^−1^, respectively, and the highest SGD-driven fluxes of chemical constituents were estimated (1.7 × 10^6^ mol day^−1^ for dissolved inorganic nitrogen, 0.1 × 10^6^ mol day^−1^ for dissolved inorganic phosphorus (DIP), 1.1 × 10^6^ mol day^−1^ for dissolved silicon, 0.5 × 10^6^ mol day^−1^ for dissolved organic carbon, 1.6 × 10^6^ mol day^−1^ for dissolved organic nitrogen, 0.4 × 10^6^ mol day^−1^ for particulate organic carbon, and 38 × 10^6^ g QS day^−1^ for FDOM_H_). SGD-driven fluxes of dissolved nutrient and organic matter were over 90% of the total input fluxes in Hwasun Bay. Our results highlight the potential of using the FDOM_H_-Si mass balance model to effectively measure SGD within a specific area (i.e., volcanic islands) under specific weather conditions (i.e., typhoon/storm). In oligotrophic oceanic regions, SGD-driven chemical fluxes from highly permeable islands considerably contribute to coastal nutrient budgets and coastal biological production.

## Introduction

Submarine groundwater discharge (SGD) comprises terrestrially derived fresh groundwater and re-circulated seawater^1–3^. SGD can be affected by tidal pumping, wave set-up, currents, and density gradients^4–6^. In particular, under strong winds of 10 m s^−1^ (i.e., storms and typhoons), wave pumping rates can increase by orders of magnitude exceeding the rates of fresh water inputs from runoff and SGD^7^. In volcanic islands, such as Hawaii (USA), Jeju Island (Korea), and Mauritius, high rates of SGD occur owing to a high relief and permeability in addition to poorly developed river drainage systems^8–10^.

SGD is an important pathway for transporting chemical constituents, such as dissolved organic matter (DOM), nutrients, radionuclides, and trace elements to the coastal ocean on a regional scale^11–15^ as well as the basin^2,16^ and global scales^17^. The chemical constituent fluxes via SGD are similar to or often much higher than those through river discharge into the coastal ocean. SGD may play an especially important role in tropical islands (e.g., Jeju Island, Hawaii, Mauritius, Balearic Islands) that are dominated by substantial precipitation and highly permeable rocks^13,18–21^. For example, in Jeju island, SGD-driven fluxes of dissolved inorganic nitrogen (DIN) and organic nitrogen (DON) in Hwasun Bay are larger than those through large rivers around the world, such as the Delaware, Colorado, and Stikine^22^. Excess nutrient inputs through SGD in Bangdu Bay and Jocheon harbor, Jeju Island, have resulted in green tides of *Ulva* spp. (*U. conglobata* and *U. pertusa*)^23–25^.

SGD is an invisible phenomenon; therefore, direct estimation is difficult. Several studies have attempted to measure SGD using hydrological processes^26,27^, seepage measurements^4,9^, and geochemical tracers such as Ra isotopes^2,3^ and ^222^Rn^5^. In addition, dissolved silicon (DSi) can be used as a geochemical tracer for determining SGD when it shows a conservative behavior in coastal aquifers^28,29^.

Fluorescent dissolved organic matter (FDOM), an active part of DOM absorbing ultraviolet and short visible light, represents considerable portions of the DOM pool in land and coastal water^30,31^. According to its origin and optical properties, there are mainly two types of FDOM, i.e., humic-like FDOM (FDOM_H_) and protein-like FDOM (FDOM_P_), in coastal environments^31^. Recent studies show that SGD could be a hidden source of FDOM in coastal oceans^22,32,33^. A previous study applied FDOM as a tracer owing to its spectral characteristics to identify groundwater-driven DOM in the coral reefs of Hawaii^34^. In Jeju Island, SGD enhanced the inventory of FDOM_H_ in coastal seawater by 2–3 times; furthermore, FDOM_H_ exhibits conservative behavior during all seasons^32^.

This study aimed to estimate SGD in a volcanic island, Jeju Island, using FDOM_H_ and Si mass balance model for the first time. Furthermore, it compares the SGD and the associated flux of nutrients and DOM before and after a typhoon.

## Results and discussion

### Origin and behavior of nutrients and organic matter in Hwasun Bay during the typhoon

The concentrations of DIN in the brackish groundwater of Hwasun Bay ranged from 30 to 280 μM on October 4 (avg.: 187 ± 78 μM, n = 27), 29 to 294 μM on October 7 (avg.: 160 ± 85 μM, n = 26), and 60 to 290 μM on October 10 (avg.: 196 ± 80 μM, n = 26), which were significantly higher than those in seawater (avg.: 2.9 ± 2.9 μM, n = 14) but lower than those in fresh groundwater (avg.: 308 ± 9 μM, n = 10) (Supplementary Fig. S1a).

The concentrations of dissolved inorganic phosphorus (DIP) in the brackish groundwater of Hwasun Bay ranged from 0.5 to 3.6 μM on October 4 (avg.: 1.1 ± 0.7 μM), 0.2 to 3.0 μM on October 7 (avg.: 1.1 ± 0.6 μM), and 0.1 to 2.4 μM on October 10 (avg.: 1.2 ± 0.6 μM), which were similar to those in seawater and fresh groundwater after the typhoon (Supplementary Fig. S1b).

The concentrations of DSi in the brackish groundwater of Hwasun Bay ranged from 44 to 186 μM on October 4 (avg.: 102 ± 41 μM), 57 to 146 μM on October 7 (avg.: 98 ± 27 μM), and 83 to 319 μM on October 10 (avg.: 163 ± 78 μM), which were significantly higher than those in seawater (avg.: 6.5 ± 2.0 μM) but lower than those in fresh groundwater (avg.: 226 ± 85 μM) (Supplementary Fig. S1c).

The concentrations of dissolved organic carbon (DOC) in the brackish groundwater of Hwasun Bay ranged from 44 to 72 μM on October 4 (avg.: 57 ± 7 μM), 26 to 87 μM on October 7 (avg.: 53 ± 14 μM), and 33 to 54 μM on October 10 (avg.: 45 ± 6 μM), which were significantly lower than those in seawater (avg.: 67 ± 4 μM) but higher than those in fresh groundwater (avg.: 31 ± 3 μM) (Supplementary Fig. S1d).

The concentrations of DON in the brackish groundwater of Hwasun Bay ranged from 24 to 132 μM on October 4 (avg.: 63 ± 32 μM), 20 to 220 μM on October 7 (avg.: 105 ± 60 μM), and 58 to 402 μM on October 10 (avg.: 202 ± 95 μM), which were significantly higher than those in seawater (avg.: 12 ± 12 μM) but lower than those in fresh groundwater (avg.: 502 ± 213 μM). DON concentrations in groundwater increased by more than 2 times after the typhoon (Supplementary Fig. S1e).

**Figure 1:**
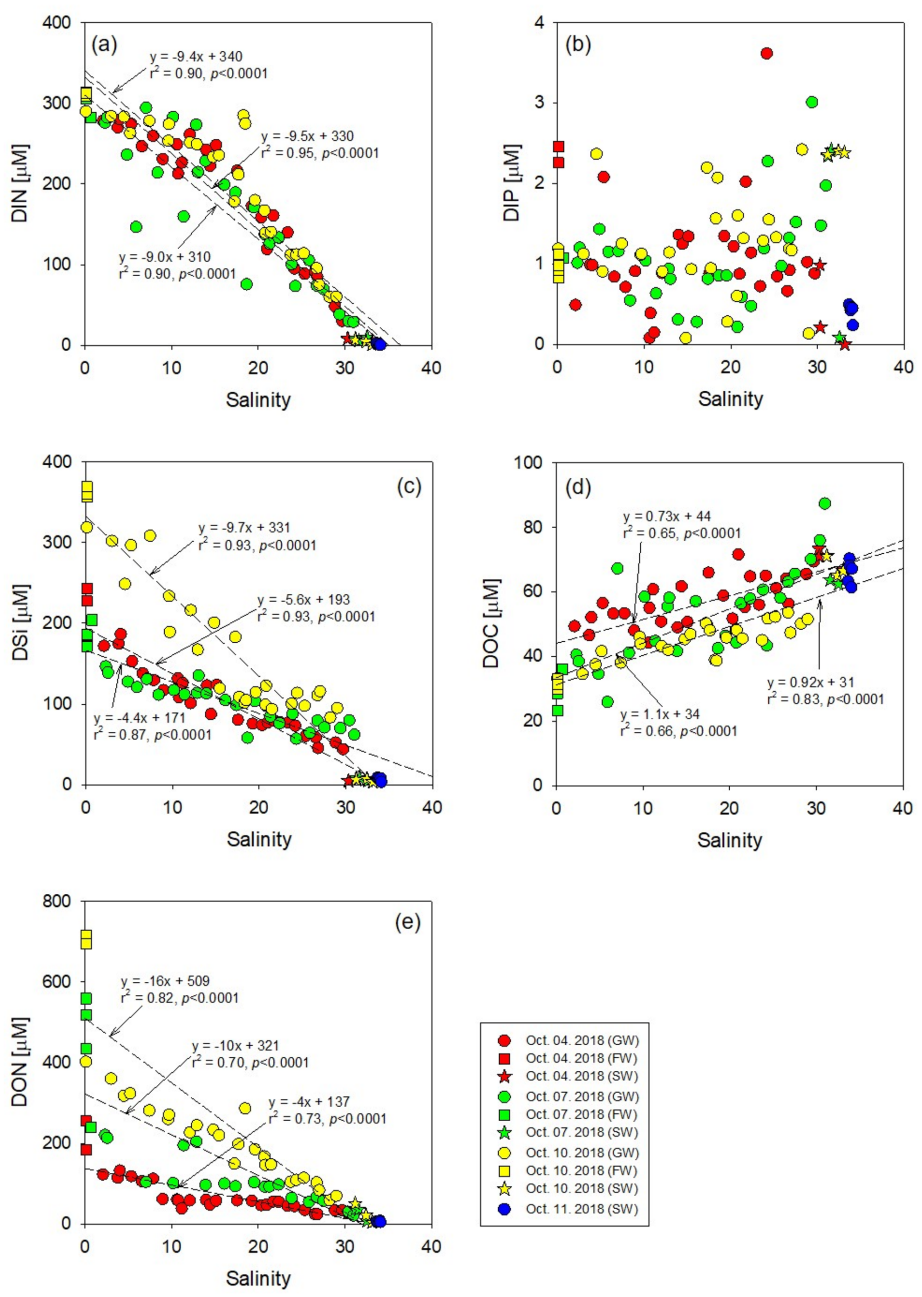
Scatter plots of (**a**) dissolved inorganic nitrogen (DIN), (**b**) dissolved inorganic phosphorus (DIP), (**c**) dissolved silicon (DSi), (**d**) dissolved organic carbon (DOC), and (**e**) nitrogen (DON) versus salinity in Hwasun Bay. Red, green, and yellow dots indicate samples collected on October 4, 2018 (before the typhoon), October 7, 2018 (one day after the typhoon), and October 10, 2018 (4 days after the typhoon) in Hwasun Bay. This figure was drawn with sigma plot software (ver. 10.0).

The plots of DIN, DSi, DOC, and DON concentrations versus salinity show conservative mixing for salinity ranging from 0 to 34 in Hwasun Bay (Fig. 1). These results indicate that the sink and source of nutrients and DOM are negligible in this bay, perhaps owing to rapid seepage rates along the coast of Jeju Island (0.14–0.82 m day^−1^)^9^. The concentrations of DIN, DIP, DSi, and DON were higher in groundwater than those in seawater, whereas DOC concentrations were lower in groundwater than those in the bay seawater during all sampling periods (Fig. 1). In addition, the average concentrations of DSi and DON in groundwater increased after the typhoon (Fig. 1). The increase in DSi appears to be due to enhanced silicate weathering rates and DON appears to originate from the soil matrix after typhoons. Previous studies reported that extreme weather, such as typhoon, induced mechanical weathering and increased sediment and soil supply to channels^35–37^.

**Figure 2:**
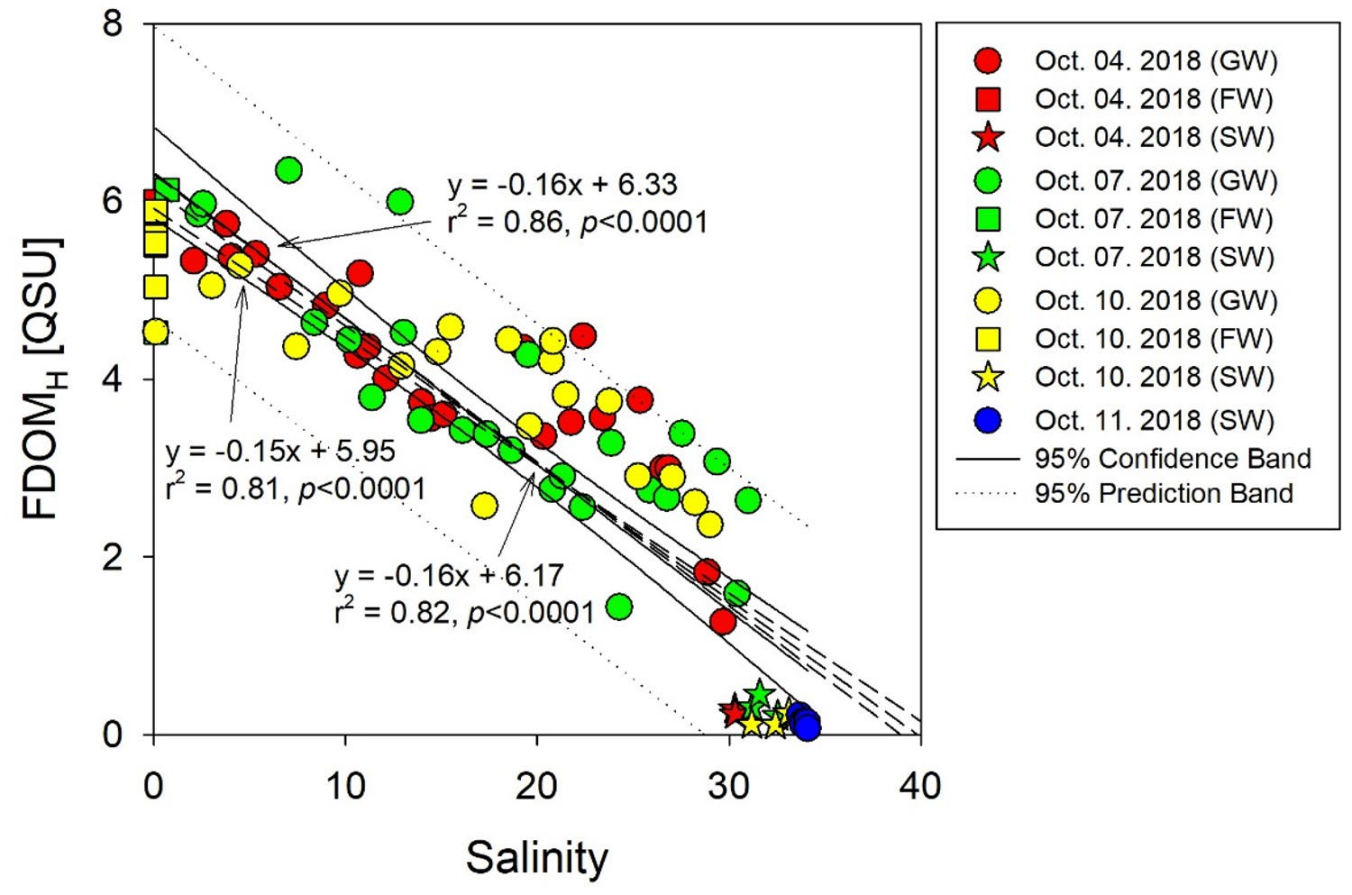
Correlation between humic-like fluorescent dissolved organic matter (FDOM_H_) and salinity of groundwater samples in Hwasun Bay during all sampling campaigns. The solid lines and dotted lines show the 95% confidence interval and 95% prediction interval for the regression line, respectively. This figure was drawn with sigma plot software (ver. 10.0).

The FDOM_H_ intensities in the brackish groundwater of Hwasun Bay ranged from 1.3 to 5.7 QSU on October 4 (avg.: 4.0 ± 1.1 QSU), 1.4 to 6.3 QSU on October 7 (avg.: 3.7 ± 1.3 QSU), and 2.4 to 5.3 QSU on October 10 (avg.: 3.9 ± 0.9 QSU), which were significantly higher than those in seawater (avg.: 0.2 ± 0.1 QSU) but lower than those in fresh groundwater (avg.: 5.6 ± 0.5 QSU) (Supplementary Fig. S1g). The FDOM_H_ intensities, indicating humic sources such as terrestrial, anthropogenic, and agricultural sources^31^, decreased with increasing salinity in all sampling campaigns (Fig. 2). However, in the high salinity zone, FDOM_H_ showed deviations from the seawater and the fresh groundwater mixing line in the subterranean estuary, which might be a result of infiltration and transformation of marine organic matter in the beach sediments during tidal inundation^12,33^. However, the non-conservative FDOM behavior in this saline zone differs from the lower salinity zone where FDOM_H_ generally behaves conservatively. This conservative behavior is highly dependent on the balance between freshwater supply rates and mixing relative to the biological production rate of FDOM.

**Figure 3:**
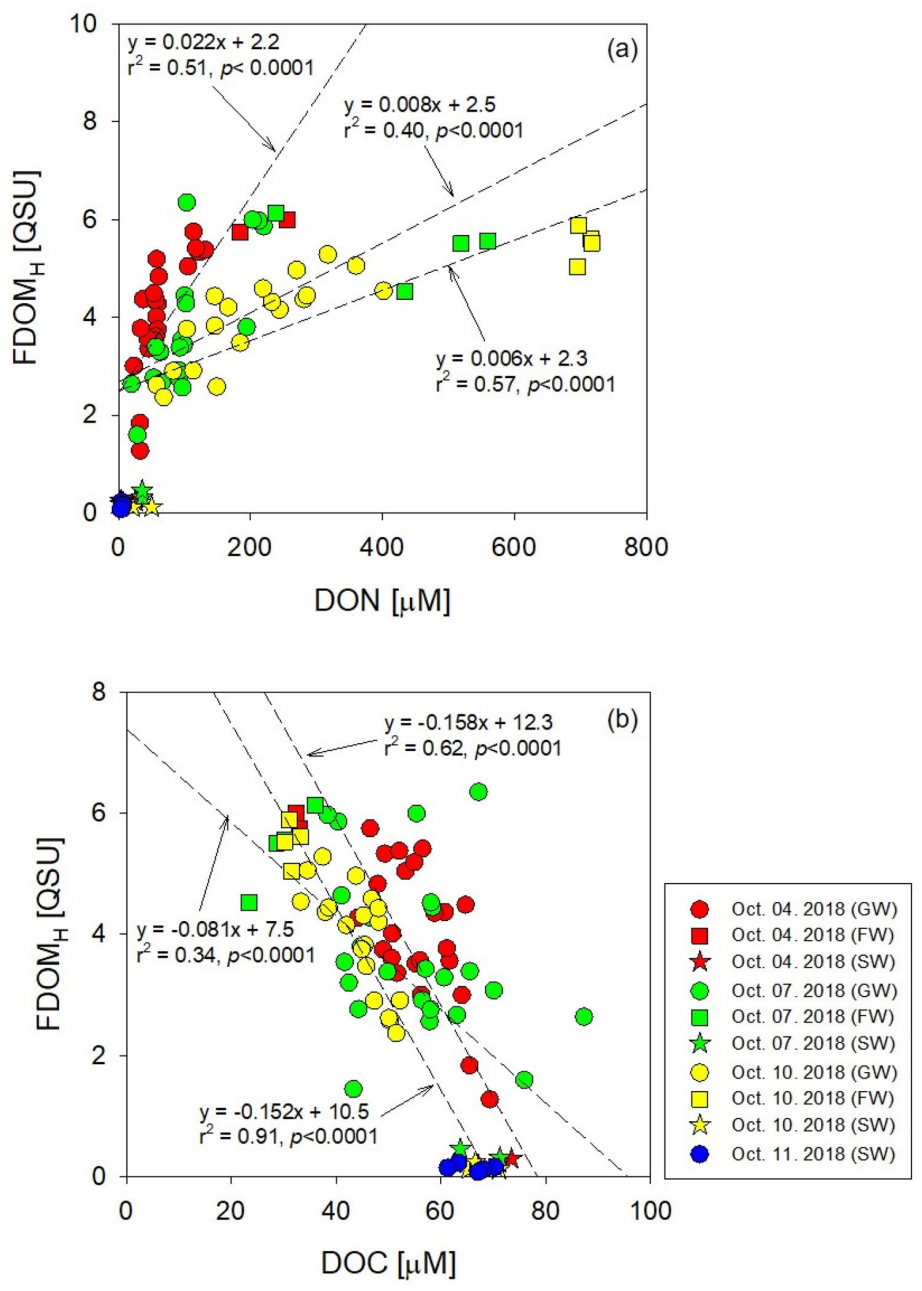
Scatter plots of humic-like fluorescent dissolved organic matter (FDOM_H_) versus (**a**) dissolved organic nitrogen (DON) and (**b**) carbon (DOC). This figure was drawn with sigma plot software (ver. 10.0).

FDOM_H_ showed good positive correlations with DON (Fig. 3a) and good negative correlations with DOC (Fig. 3b). Coble^38^ reported similar correlations between DOC and DON and peak C for the “humic-like” component, which were observed in all seasons in Hwasun Bay^22^. These high concentrations of nutrients and DON in groundwater can be mainly attributed to terrestrial sources, including agricultural activity or domestic wastewater, whereas DOC showed positive correlations with salinity, implying a marine origin.

The concentrations of particulate organic carbon (POC) in the brackish groundwater of Hwasun Bay ranged from 6.0 to 56 μM on October 4 (avg.: 21 ± 13 μM), 4 to 73 μM on October 7 (avg.: 31 ± 20 μM), and 5.0 to 40 μM on October 10 (avg.: 20 ± 14 μM), which were lower than those in seawater (avg.: 36 ± 30 μM) but higher than those in fresh groundwater (avg.: 11 ± 10 μM) except for one day after the typhoon. Exceptionally high concentrations of POC in fresh groundwater were observed during the typhoon (avg: 83 ± 33 μM) (Supplementary Fig. S1f). This result shows that short-term episodic storm events could increase current sediment delivery loads, thereby increasing POC delivery in volcanic islands.

### Estimating SGD in Hwasun Bay using FDOM_H_ and Si mass balance models

Ra isotopes and ^222^Rn have served as the most powerful tools for gauging the magnitude and mechanism of SGD because Ra isotopes and ^222^Rn are chemically conservative in seawater and enriched in groundwater^2,39–41^. Although it has received less attention than radioisotope tracers, DSi is also a useful tracer for determining SGD when DSi is highly enriched in fresh groundwater and it shows a conservative behavior in coastal aquifers^28,29^. In this study, the concentrations of DSi in fresh groundwater showed the highest values in fresh groundwater samples and exhibited good negative linear correlations with salinity. Thus, DSi is expected to be a good tracer for estimating SGD flux.

In this study, we applied FDOM_H_ as a tracer to estimate SGD flux. Although DOM characteristics vary depending upon the various environments and FDOM is known to account for 20–70% of DOM (generally represented by DOC), it has the highest values in coastal regions, where freshwater inputs are dominant^31^. In this study area, FDOM_H_ showed strong negative correlation with salinity and overwhelmed by the overall dilution of the terrestrial FDOM_H_ relative to the internal production rate of FDOM. Thus, it has been used as a good SGD tracer in areas with high SGD rate in volcanic islands^32,34^. All previous studies conducted in Jeju Island consistently showed good negative correlation between FDOM_H_ and salinity^22,32^, which indicates apparent recalcitrant FDOM_H_ sources from terrestrial inputs. Thus, we assumed that terrestrial origin FDOM_H_ behaves conservatively in the subterranean estuary, and fresh groundwater flux was calculated using FDOM_H_ as a tracer also with DSi. In the steady state, the mass balance of FDOM_H_ and Si could be expressed as follows:

1 $$F_{Diff}^{{\text{FDOM}}_{\text{H}}} + \;C_{GW}^{{\text{FDOM}}_{\text{H}}} \times A_{Bott} \times \;\psi_{SGD} \; - C_{EX}^{{\text{FDOM}}_{\text{H}}} \times V_{S} \times\uplambda _{Mix\;} = 0$$

2 $$F_{Diff}^{{{\text{Si}}}} + \;C_{GW}^{{{\text{Si}}}} \times A_{Bott} \times \;\psi_{SGD} \; - C_{EX}^{{{\text{Si}}}} \times V_{S} \times\uplambda _{Mix\;} = 0$$

where the terms on the left side of the equation indicate input fluxes arising from diffusion from sediments (first term) and submarine groundwater flow (second term) and mixing with open ocean water (third term).

The diffusion from sediments was calculated for an area (1.90 × 10^7^ m^2^) in Hwasun Bay via the regeneration rates of FDOM_H_ (1.4 × 10^5^ μg QS m^−2^ day^−1^) and Si (5 mmol m^−2^ day^−1^) in sediments^42,43^. SGD flux was calculated based on average concentrations of FDOM_H_ (4.03 ± 1.13 g QS m^−3^ on October 4, 3.68 ± 1.34 g QS m^−3^ on October 7, and 3.93 ± 0.89 g QS m^−3^ on October 10) and Si (102 ± 41 mmol m^−3^ on October 4, 98 ± 27 mmol m^−3^ on October 7, and 163 ± 78 mmol m^−3^ on October 10) in groundwater, an area of the bay, and the unknown seepage rate of groundwater (m day^−1^).

Mixing with open ocean water was evaluated based on the differences in concentration between bay seawater and open ocean water for FDOM_H_ (0.12 ± 0.06 g QS m^−3^ on October 4, 0.14 ± 0.12 g QS m^−3^ on October 7, and 0.08 ± 0.06 g QS m^−3^ on October 10) and Si (3 ± 2 mmol m^−3^ on October 4, 4 ± 1 mmol m^−3^ on October 7, and 4 ± 2 mmol m^−3^ on October 10), the water volume of the bay, and the unknown exchange rate between bay seawater and open ocean water.

We estimated the seepage rate of groundwater and water residence times simultaneously by solving Eqs. (1 and 2) for the unknown SGD (Ψ_SGD_) terms and water residence time (T_W_ = 1/λ_Mix_). The water residence time and seepage rate of groundwater calculated using these simultaneous equations were 0.9, 1.0, and 1.0 days with uncertainties of approximately 90% and 0.30, 0.51, and 0.26 m day^−1^ with uncertainties of approximately 105% on October 4, 7, and 10, respectively. The large uncertainties show that the FDOM_H_ deviations of both the seawater and the freshwater end member mixing-line may not be determinant in this calculation. In addition, our estimation already included the uncertainty of FDOM_H_ intensities in groundwater samples. The water residence time in this study was slightly lower than that (2.5 days) calculated using the tidal prism model, whereas the seepage rate of groundwater in this study was relatively higher than that (0.12 m day^−1^) obtained using the ^222^Rn-Si mass balance model^13^ although this study was conducted on the same survey area as that employed by Kim et al.^13^. In general, the main driving forces of SGD were affected by hydraulic gradients between the land and ocean, tidal and wave pumping, convection-driven processes, and precipitation^1,5,44^. In particular, during storms and typhoons, wave pumping rates can increase by orders of magnitude exceeding the rates of fresh water inputs from runoff and SGD^7^. According to the Korea Meteorological Administration (KMA, https://web.kma.go.kr/eng/index.jsp), the amount of rainfall three days before each sampling campaign in Hwasun Bay was 3.4, 337, and 0 mm on October 4, 7, and 10, respectively. Thus, the difference in the seepage rate of groundwater appears to be associated with heavy rainfall and wave pumping arising from the typhoon and/or the uncertainties associated with different methods. Previous studies have reported that there were approximately 50% to > 100% uncertainties associated with SGD estimation using ^222^Rn and Ra tracers^45–47^ owing to the natural variability of isotope tracers in the groundwater endmember and loss by mixing with outer-bay water in coastal regions.

### SGD-driven nutrient and organic matter fluxes in Hwasun Bay

The SGD-driven fluxes of DIN, DIP, DSi, DOC, DON, POC, and FDOM_H_ were calculated by multiplying the average concentration with uncertainty in groundwater by the SGD (5.7 × 10^6^ m^3^ day^−1^ on October 4, 9.7 × 10^6^ m^3^ day^−1^ on October 7, and 4.9 × 10^6^ m^3^ day^−1^ on October 10 with combined uncertainties of 105%) using the FDOM_H_-Si mass balance model for Hwasun Bay (Table 1). In this calculation, DIP showed non-conservative behavior and determined which concentrations were highly dispersed with the highest uncertainties (~ 57%). The highest SGD-driven fluxes of nutrients and DOM were obtained one day after the typhoon and were higher than those (0.3 × 10^6^ mol day^−1^ for DIN, 0.003 × 10^6^ mol day^−1^ for DIP, 0.2 × 10^6^ mol day^−1^ for DSi, 0.1 × 10^6^ mol day^−1^ for DOC, and 0.1 × 10^6^ mol day^−1^ for DON) reported by Kim et al.^13^ and Kim et al.^22^.

**Table 1:** Fluxes of dissolved inorganic nutrients and organic matter via SGD in Hwasun Bay of Jeju Island during each sampling campaign.

Sampling date	SGD flux (× 10^6^ m^3^ day^−1^)	Fluxes						
		× 10^6^ mol day^−1^						× 10^6^ g QS day^−1^
		DIN	DIP	DSi	DOC	DON	POC	FDOM_H_
4th October, 2018 (before the typhoon)	5.7	1.1	0.07	0.5	0.3	0.4	0.1	24
7th October, 2018 (one day after the typhoon)	9.7	1.7	0.1	1.1	0.5	1.6	0.4	38
10th October, 2018 (4 days after the typhoon)	4.9	1.0	0.06	0.8	0.2	1.4	0.1	21

The calculated SGD-derived nutrient and organic matter fluxes have combined uncertainties ranged from 110 to 125%.

The input fluxes of nutrients can be attributed to diffusion from bottom sediments as well as SGD in Hwasun Bay. The diffusion fluxes of DIN, DIP, DSi, DOC, and DON from bottom sediments were calculated by multiplying the area of Hwasun Bay by the previously reported rates of regeneration of nutrients and diffusive DOM fluxes from bottom sediments^42,48–50^. The estimated diffusion fluxes of DIN, DIP, DSi, DOC, and DON from bottom sediments were approximately 0.03 × 10^6^, 0.01 × 10^6^, 0.10 × 10^6^, 0.04 × 10^6^, and 0.004 × 10^6^ mol day^−1^, respectively. The fluxes of DIN, DIP, DSi, DOC, and DON through SGD, based on the overall nutrient fluxes into the bay, contribute approximately 98%, 88%, 89%, 89%, and 100% of the total fluxes, respectively. Thus, SGD appears to be an important pathway as a nutrient and DOM source in Hwasun Bay.

The fluxes of DOC through SGD in Hwasun Bay were one order of magnitude higher than diffusion fluxes of DOC and higher than the fluxes of POC through SGD (Table 1). This result indicates that SGD-derived DOC in Hwasun Bay could be the most important source of carbon. However, in situ production by biological activities rather than by SGD in this bay may play an important role in determining the carbon budget, which can be confirmed by the concentrations of DOC in groundwater being lower than those in the bay seawater and the negative correlations between DOC and FDOM_H_ (Fig. 3b).

The fluxes of FDOM_H_ through SGD (24 × 10^6^ g QS day^−1^ on October 4, 38 × 10^6^ g QS day^−1^ on October 7, and 21 × 10^6^ g QS day^−1^ on October 10 with combined uncertainties of ~ 110%; Table 1) in Hwasun Bay were one order of magnitude higher than the diffusion fluxes of FDOM_H_ and two orders of magnitude higher than those in Jochun Bay^32^ (northern part of Jeju Island; 0.1 ~ 0.4 × 10^6^ g QS day^−1^) owing to the relatively low SGD flux (4.1–6.9 × 10^4^ m^3^ day^−1^), calculated using the ^222^Rn mass balance model^25^. The fluxes of FDOM_H_ through SGD into the bay contribute approximately 80% of the total input fluxes. These results highlight the possibility of SGD being an important hidden source of FDOM_H_ in the volcanic island. In oligotrophic oceanic regions, coral reefs are highly productive ecosystems that should be protected from the damaging effects of solar UV radiation^51^. Thus, SGD-derived FDOM_H_ could be beneficial to the sustenance of coral ecosystems considering their ability to protect coral reefs from bleaching under harmful UV radiation in surface water^51,52^. In the last three decades, seaweeds have replaced corals, leading to the global decline of coral reefs in association with ocean acidification and changing nutrient dynamics^53^. However, high loads of FDOM_H_ arising from SGD provides favorable conditions for coral ecology.

## Conclusions

The seepage rate of groundwater estimated using an FDOM_H_-Si mass balance model was approximately 2–4 times higher than that estimated using the ^222^Rn mass balance model reported by Kim et al.^13^. This difference may be attributable to the high level of rainfall and wave pumping owing to the typhoon rather than uncertainties associated with the use of each method. Owing to its several advantages, including relative simplicity, low cost, chemical conservativeness in seawater, and enrichment in groundwater relative to seawater, the FDOM_H_-Si mass balance model can be effective for estimating SGD in coastal areas of a highly permeable zone without any continuous river or stream discharge. The larger SGD-driven nutrient, DOM, and FDOM_H_ fluxes in Hwasun Bay during typhoons could play an important role in biogeochemistry linked to oceanic production and carbon fluxes. Nevertheless, more extensive observations are necessary to evaluate SGD and nutrient fluxes through SGD depending on geophysical processes.

## Materials and methods

### Study area and typhoon information

Jeju Island, a volcanic island (area of ~ 1830 km^2^), is located in the southern sea of Korea and has a shield volcano named Mountain Halla, with an elevation of 1950 m. The island is composed mainly of basaltic rocks formed by Cenozoic volcanism. Therefore, although it experiences high rainfall (1140–1960 mm year^−1^)^54,55^, sustained stream flow is rare. According to hydrologic budget analyses, approximately 50% of the total precipitation (1.5 × 10^9^ m^3^ year^−1^) contributes to groundwater recharge^54,56^.

The study site, Hwasun Bay (area of ~ 19 km^2^ and a mean depth of ~ 13 m), is located in the southwestern part of Jeju Island. The residence time of bay seawater, estimated by the tidal prism method, is approximately 2.5 days^13^. With the absence of continuous rivers or stream discharge into the bay, SGD plays a dominant role in the transport of terrestrial nutrients, DOM, and trace elements to the ocean^13,21–23,57,58^.

Typhoon Kong-rey, the 25th typhoon of the year, occurred on 28 September 2018. When the typhoon approached Jeju Island on October 6, it was categorized as a medium-scale typhoon with a central pressure of 975 hPa, maximum wind speed of 32 m/s, and strong wind radius of 350 km; furthermore, it had a cumulative precipitation reaching 718.5 mm (KMA, https://web.kma.go.kr/eng/index.jsp).

### Sampling

**Figure 4:**
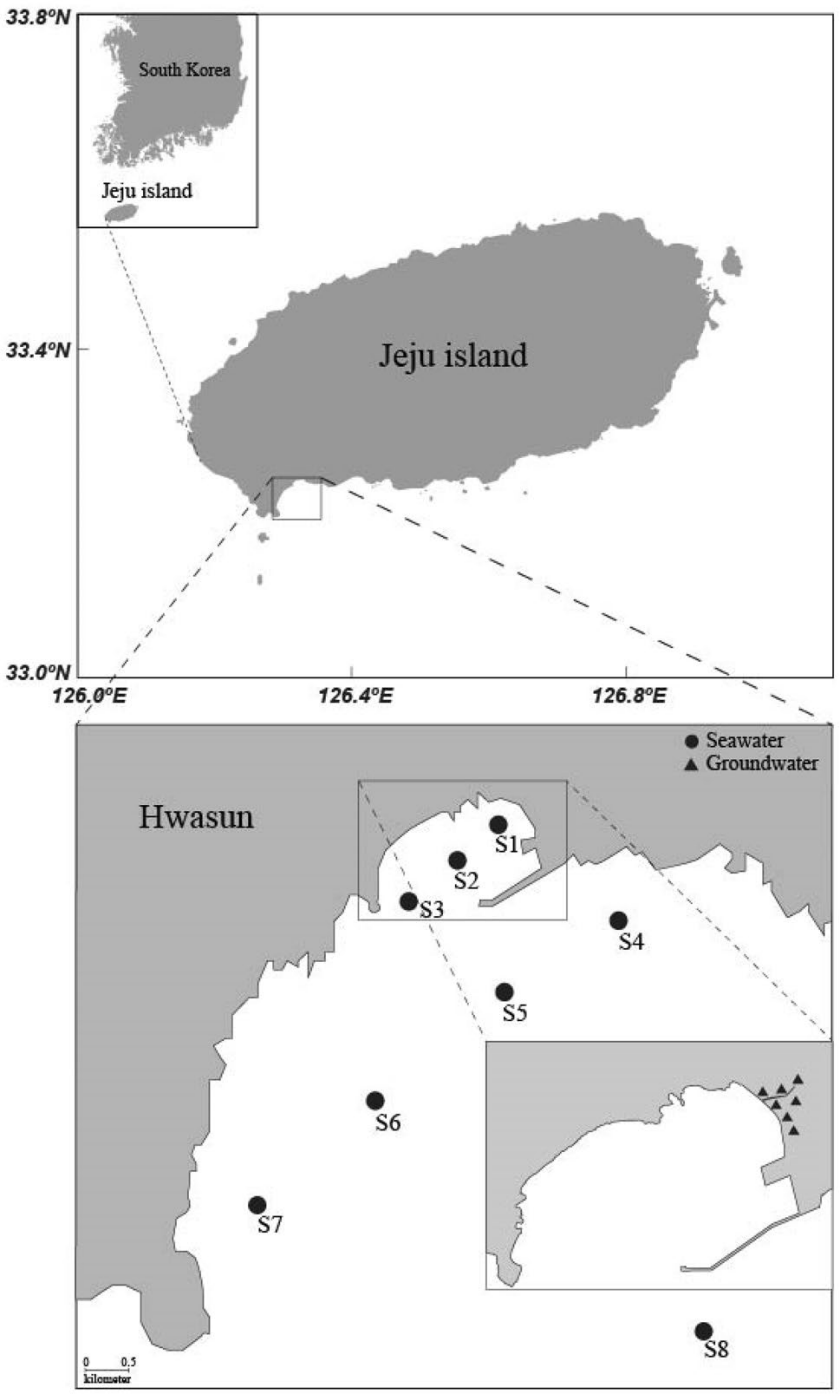
A map showing the study site, Hwasun Bay of Jeju Island, and sampling locations of seawater (circle) and groundwater (triangle) in October 2018. This figure was drawn using Adobe Illustrator Version 24.1.3.

Samples of fresh groundwater, brackish groundwater, and coastal seawater of the inner bay (S1, S2, and S3) were collected on October 4 (before the typhoon), October 7 (one day after the typhoon), and October 10 (4 days after the typhoon) in 2018 in Hwasun Bay to analyze dissolved inorganic nutrients (NO_3_^−^, NO_2_^−^, NH_4_^+^, Si(OH)_4_, and PO_4_^3−^), DOC and DON, and FDOM (Fig. 4). Seawater samples from the outer bay were collected from five stations (S4–S8) on October 11, 2018 because sampling surveys could not be conducted in the outer bay during the sampling period because of the typhoon. Fresh groundwater samples were collected from groundwater wells along the coastline. Brackish groundwater samples were collected from shallow pits dug into nearshore sandy sediments above porous basaltic rocks. Seawater samples from the inner bay were collected in the low-tide line using a plastic beaker. Seawater samples from the outer bay were collected in Niskin bottles attached to a CTD rosette on the R/V *A-Ra* of Jeju National University, Korea.

Water samples for FDOM, DOC, total dissolved nitrogen (TDN), and dissolved nutrients analyses were collected and filtered immediately in the field using a Whatman 0.7 μm disposable syringe filter. FDOM samples were stored in pre-combusted amber glass vials and kept refrigerated (< 4 °C) until analysis. The subsamples for DOC and TDN were transferred into pre-combusted glass ampoules (in the furnace at 500 °C for 4 h) and acidified with 6 M HCl (pH ~ 2); the ampoules were then flame sealed. The subsamples for dissolved inorganic nutrients were stored in HDPE bottles (Nalgene) and frozen until analysis. Samples for POC were collected in 1-L of HDPE bottle and were filtered through a 0.7 μm GF/F filter. The filter papers were placed in a petri dish and frozen for storage.

### Analytical methods

Salinity was measured in situ using an YSI Pro Series conductivity probe. Fluorescence measurements of FDOM were conducted using a spectrofluorometer (SCINCO FluoroMate FS-2) in the scan mode. Emission (Em) spectra (250–500 nm) were collected at 2 nm intervals at excitation (Ex) wavelengths of 250–360 nm (5 nm intervals). Water Raman scattering was eliminated by subtracting the daily fresh distilled water signals from the sample data. Data intensities, obtained in counts per second (cps), were normalized with quinine sulfate standards (fluorescence spectra of quinine sulfate standard solution in 0.1 N H_2_SO_4_ at Ex/Em of 350/450 nm) and expressed as parts per billion of quinine sulfate equivalents (ppb QSE). Excitation-emission matrices (EEMs) for all data with smoothing were obtained using MATLAB with Savitzky-Goray filters. The PARAFAC model was applied to our 3D EEMS data and validated using split-half analysis and core consistency test^59^. Three components were statistically identified as component 1 (Ex_max_/Em_max_ = 300/370 nm), component 2 (Ex_max_/Em_max_ = 315/340 nm), and component 3 (Ex_max_/Em_max_ = 340/428 nm) (Supplementary Fig. S2). According to Coble^31^, component 1 and 3 are indicative of marine FDOM_H_ (peak M) and terrestrial FDOM_H_ (peak C), respectively, and component 2 is found to be a FDOM_P_ (peak T).

Inorganic nutrients, including NO_3_^−^, NO_2_^−^, NH_4_^+^, Si(OH)_4_, and PO_4_^3−^ were analyzed using a nutrient auto-analyzer (Alliance Instruments, FUTURA II +). In this study, we define DIN as the sum of NO_3_^−^, NO_2_^−^, and NH_4_^+^. Artificial seawater (salinity: 35) was used as the matrix for the blank and standard. The analytical uncertainties were within 2% for DIN, DIP, and DSi according to certified reference materials (MOOS-1 from National Research Council, Canada and DSR from University of Miami, USA).

DOC and TDN concentrations were analyzed using a TOC-V_CPH_ analyzer (Shimadzu, Japan). Based on the calibration curves of acetanilide (C:N = 8), DOC and TDN measurements were standardized. The measured values of 44 μmol L^−1^ for DOC (n = 6) and were 32 μmol L^−1^ for TDN (n = 6) agreed well within 5% for the certified values. DON concentrations were calculated by subtracting the DIN concentrations from TDN concentrations.

Prior to analyzing POC, filter papers were dried at 50 °C for 12 h to remove moisture from the filter paper. Then, acid fumigation was conducted with HCl for 12 h in a desiccator to remove inorganic carbon. POC concentration was measured using a Thermo Scientific Flash 2000 element analyzer.

## Supplementary Information

Supplementary Information.
