# Supplementary material for: Estimating submarine groundwater discharge in Jeju volcanic island (Korea) during a typhoon (Kong-rey) using humic-fluorescent dissolved organic matter-Si mass balance
Source: Sci Rep. 2021 Jan 13;11:941. doi: 10.1038/s41598-020-79381-0 (PMC7807084; doi:10.1038/s41598-020-79381-0)
Supplement: Supplementary file 1 — Supplementary Information. [file 41598_2020_79381_MOESM1_ESM.docx]

**Supplementary Information**

**Estimating submarine groundwater discharge in Jeju volcanic island (Korea) during a typhoon (Kong-rey) using humic-fluorescent dissolved organic matter-Si mass balance**

*Hyung-Mi Cho^1^, Tae-Hoon Kim^2^*, Jae Hong Moon^3^, Byung Chan Song^3^, Dong-Woon Hwang^4^, Taejin Kim^5^, Dong-Hoon Im^4^*

*^1^Department of Ocean Sciences, Inha University, 100 Inha-ro, Incheon 22212, Republic of Korea*

^2^*Department of Oceanography, Faculty of Earth Systems and Environmental Sciences, Chonnam National University, Gwangju 61186, Republic of Korea*

^3^*Department of Earth and Marine Sciences, Jeju National University, Jeju, 63243, Republic of Korea.*

*^4^Marine Environment Research Division, National Institute of Fisheries Science, Busan 46083, Republic of Korea*

*^5^Department of Oceanography, Pukyong National University, 45 Yongso-ro, Nam-gu, Busan 48513, Republic of Korea*

**
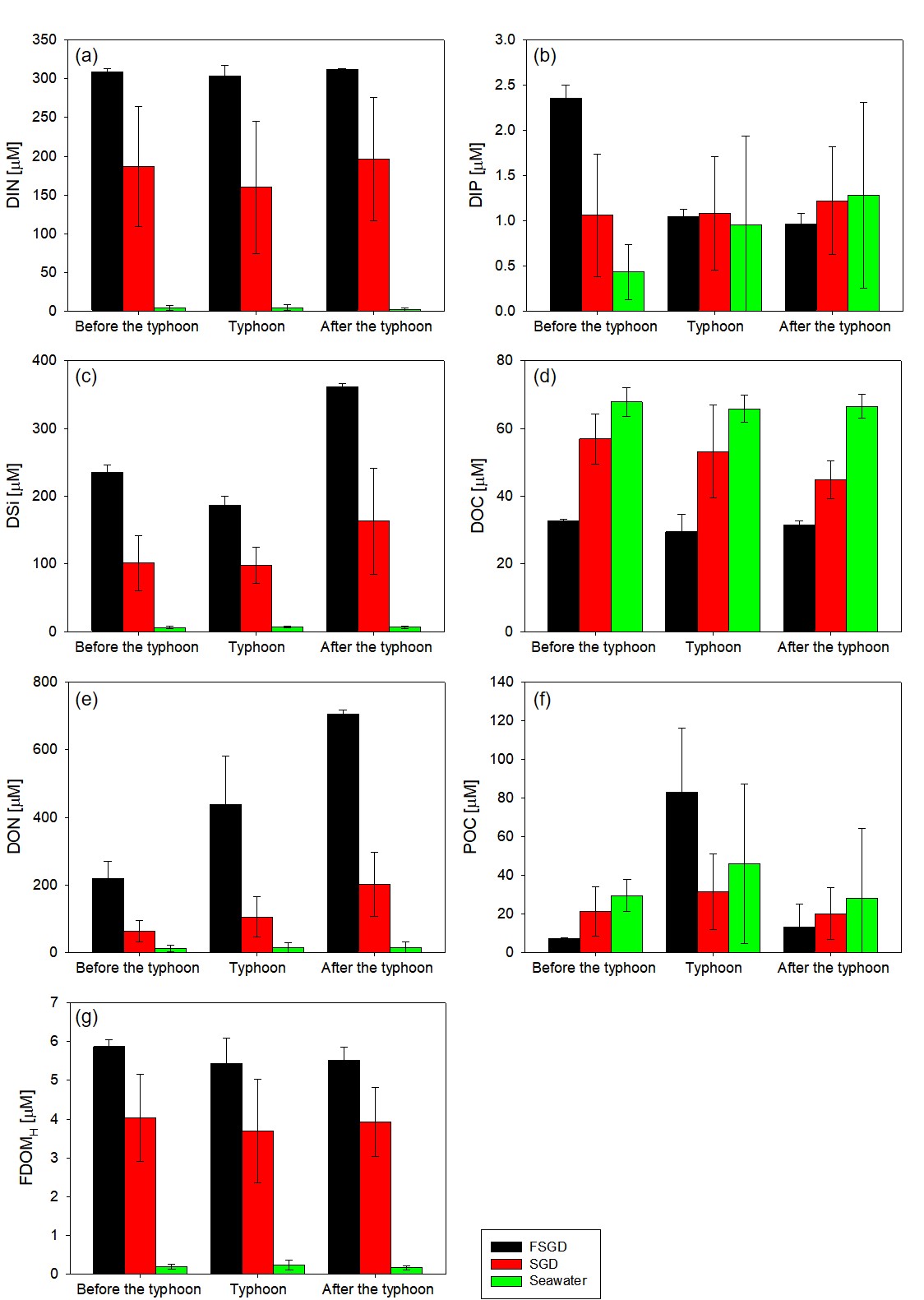
**

**Figure S1**

Bar graphs with error bars showing average values and standard deviations of (a) dissolved inorganic nitrogen (DIN), (b) dissolved inorganic phosphorus (DIP), (c) dissolved silicon (DSi), (d) dissolved organic carbon (DOC) and (e) nitrogen (DON), (f) particulate organic carbon (POC), and (g) humic-like fluorescent dissolved organic matter (FDOM_H_) of fresh groundwater (FSGD; black), saline groundwater (SGD; red), and seawater (green) in Hwasun Bay during each sampling campaign (before the typhoon, during the typhoon, and after the typhoon). This figure was drawn with sigma plot software (ver. 10.0).


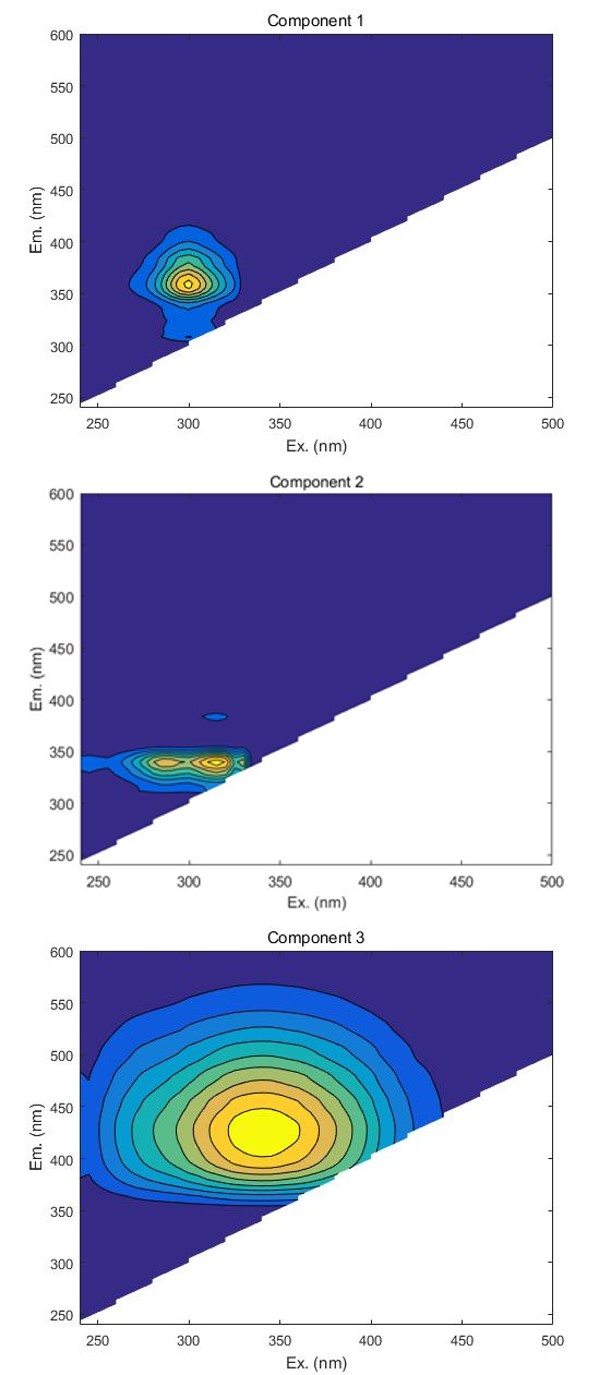


**Figure S2**

Excitation-emission matrix spectroscopy (EEMs) of the three components.
